# Functional quality evaluation and shelf life study of synbiotic yacon juice

**DOI:** 10.1002/fsn3.1440

**Published:** 2020-02-10

**Authors:** Sagar Dahal, Pravin Ojha, Tika Bahadur Karki

**Affiliations:** ^1^ Department of Food Technology National College of Food Science and Technology Kathmandu Nepal; ^2^ Food Research Division Nepal Agricultural Research Council Lalitpur Nepal; ^3^ Department of Biotechnology Kathmandu University Dhulikhel Nepal

**Keywords:** antioxidant activity, *Lactobacillus acidophilus*, synbiotic, yacon

## Abstract

The research aimed to utilize the prebiotic potential of yacon fruit by preparation of synbiotic yacon juice containing probiotic microorganism *Lactobacillus acidophilus* (La5) and studying the changes in physicochemical properties during storage. The fresh juice extracted was added with probiotic microorganism and was subjected for storage at refrigerated (4°C) and room condition (25°C) along with control (nonfermented) juice. The viability of probiotic strains in the juice was found to be at the satisfactory level (10^6^ CFU/ml) for 27 and 15 days, respectively, in refrigerated and room condition. Total soluble solid, pH, acidity, bioactive compounds (ascorbic acid, carotenoid content, and total phenol), and antioxidant capacity of the products were evaluated. Total soluble solid of the prepared synbiotic juice stored at refrigerated and nonrefrigerated conditions decreased from 7.8 ± 0.05 to 5.5 ± 0.26 and 5.3 ± 0.10 while the acidity increased from 0.067 ± 0.05 to 0.80 ± 0.06 and 1.02 ± 0.04 after 27 and 15 days of storage, respectively. Similarly, yeast and mold count, total plate count, and the coliform count were determined during the storage period. The low amount of yeast and mold count and absence of coliform in the synbiotic juice even after 27 and 15 days of storage indicated that the synbiotic juice can be continued to be consumed as a normal juice even after the viability decreased below the satisfactory level. Ascorbic acid, phenolic content, and antioxidant activity were higher in fermented juice when compared to control juice stored at the refrigerated conditions for the same period (*p* < .05). High therapeutic properties and numerous health benefits of yacon, when combined with the health benefits of probiotic bacteria, could lead to the development of commercial beverages with high health and nutritional values.

## Graphical abstract

The research aimed to utilize the prebiotic potential of Yacon fruit by preparation of synbiotic Yacon juice and studying the changes during storage. Ascorbic acid, phenolic content, and antioxidant activity were higher in fermented juice when compared to control juice. Functional component was also better in synbiotic yacon juice as compared to nonfermented juice.

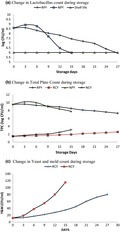

## 1 INTRODUCTION

Probiotics can be defined as live microorganisms that when administered in adequate amounts confer a health benefit on the host (Hill et al., 2014), while prebiotics are defined as “selectively fermented ingredients that allow specific changes both in composition and/or activity in the GI microflora that confer benefits upon host well‐being and health” (Gibson et al., 2017). Prebiotic can be added to food or combined with a probiotic to make synbiotic (Hamilton‐miller, 2004). A synbiotic can be defined as the supplement which contains both prebiotic and probiotic factors that work together and improve the "friendly flora" of the human intestine (Thakur, 2016).

Dairy products such as yogurts, fermented sour milk, and cheese are the major probiotic foods produced and used at present (Ranadheera, Naumovski, & Ajlouni, 2018). However, the increase in the number of vegetarian consumers has increased the demand for nondairy probiotic products (Vasudha & Mishra, 2013). Lactose intolerance, allergies to milk protein, and high cholesterol are other major drawbacks related to fermented dairy products (Granato, Branco, Nazzaro, Cruz, & Faria, 2010; Martins et al., 2013).

Yacon (*Smallanthus sonchifolius*) is a native Andean plant, grown for its juicy tuberous roots in South America, on the eastern slope of Andes from Venezuela to northwest Argentina (Valentova, Cvak, Muck, Ulrichova, & Simanek, 2003; Zardini, 1991). The presence of nondigestible oligosaccharides (NDOs) such as inulin and fructo‐oligosaccharides as well as the high amount of phenolic compounds has increased the global interest on yacon (Choque Delgado, da Silva Cunha Tamashiro, Maróstica Junior, & Pastore, 2013). Yacon stores FOS instead of starch, which is considered prebiotic as these compounds are fermented selectively by bacteria (bifidobacteria and lactobacilli) in the bowel but are neither absorbed nor hydrolyzed in the upper part of the gastrointestinal tract (Ojansivu, Ferreira, & Salminen, 2011; Pedreschi, Campos, Noratto, Chirinos, & Cisneros‐Zevallos, 2003). Besides, yacon roots have numerous health‐promoting properties including prebiotic, antidiabetic, antioxidative, and antimicrobial effects (Ojansivu et al., 2011). A study on yacon flour and its effect on intestinal microbiota and gut microflora using mice model showed stimulation in the growth of bifidobacteria and lactobacilli. The study also showed improvement in the intestinal immune system with an increase in IgA and different cytokines (Bibas Bonet et al., 2010).

The product prepared from yacon (prebiotic source) can act as a perfect alternative to the dairy probiotic products. As there is a high demand for functional food across the globe, the inclusion of yacon as a source of fructan‐type prebiotics in the production of synbiotic beverage for human use represents a great opportunity for both innovation and adding value in the functional food industry.

The objective of this research work was to prepare a yacon‐based synbiotic juice and to access the viability of probiotic microorganism in the produced beverage. The research also aims for the comparative study of bioactive compounds and antioxidant activity of fermented and nonfermented juice stored at refrigeration temperature.

## 2 MATERIALS AND METHODS

### 2.1 Chemicals and reagents

2,2‐diphenyl‐1‐picrylhydrazyl (DPPH) was purchased from Sigma‐Aldrich, Germany. Folin–Ciocalteau reagent was purchased from Merck Specialities Private Limited, India. Gallic acid was purchased from LOBA Chemie, India. Methanol was purchased from Fisher scientific, India. PCA agar, PDA agar, MRS agar, and VRBA agar media were purchased from Hi‐Media Laboratories, India. The spectrophotometer used was of Model GENESYSTM 10S Vis spectrophotometer from Thermo Scientific TM, Germany.

### 2.2 Preparation of probiotic yacon juice

Matured yacon fruit (10 days after harvesting stored at normal room temperature) was taken. Yacon fruit was peeled and cut into chunks of small size after washing in running tap water. The juice was extracted from blanched chunks (95°C for 3 min) (Reina et al., 2015). The blanching step was done to inactivate polyphenol oxidase enzyme as well as to reduce the number of indigenous yeast (Reina et al., 2015). The juice was extracted with the addition of water (0.5 times the weight of fruit), and the resulting juice was filtered through muslin clothes. The juice was then pasteurized at 85°C for 10 min for the preparation of pasteurized yacon juice.

Probiotic culture (*Lactobacillus acidophilus*, La5) was obtained from Dairy Development Corporation (DDC), Nepal. The strain was subcultured in MRS (de Mann Rogosa and Sharpe agar) media containing 1% calcium carbonate. A total of 7.00 log CFU/ml of probiotic culture was inoculated in the fermented juice and left for incubation at 37°C for 24 hr under strictly anaerobic conditions.

Probiotic and control (nonfermented) juice were dispensed into a sterile bottle and plugged tightly. Samples were then coded as NCY (Normal Control Yacon), RCY(Refrigerated Control Yacon), NPY(Normal Probiotic Yacon), and RPY (Refrigerated Probiotic Yacon) where normal juice was stored at room temperature 25°C while refrigerated juice was stored in 4°C.

### 2.3 Physicochemical analysis of control and fermented product

Total soluble solid, pH, and acidity were measured by standard techniques (Ranganna, 2007).

Lane and Eynon method using carrez solutions as a clarifying agent was used for the determination of total sugar and reducing sugar (AOAC, 2005). Clarity was measured by measuring transmittance at a wavelength of 590 nm using a UV–Vis spectrophotometer (Singh, Kumar, & Sharma, 2012).

### 2.4 Microbial analysis

For microbial analysis, 1 ml of pasteurized and fermented sample was diluted with 10 ml of saline solution and filtered through sterile Whatmann No. 1 filter paper to remove solid particles. One ml of the filtrate was used for inoculation. Different selective media were used for isolation and enumeration of different microorganisms. Potato Dextrose Agar (PDA), de Mann Rogosa and Sharpe (MRS), Violet Red Bile Agar (VRBA), and Plate Count Agar (PCA) were used for enumeration of fungi, lactobacilli, coliform, and total plate count, respectively. Three samples were used and surface plates were made in triplicates in the appropriate selective media. Pour plate method and serial dilution technique were used. For computation, the average number per plate was divided by sample volume and is expressed as CFU/ml (Sharma, 2006).

#### 2.4.1 Extract preparation

For ascorbic acid, 10–20 ml of the sample was taken and volume was made to 100 ml using 3% HPO_3_ followed by filtration.

Ascorbic acid and carotenoid content were determined as per Ranganna, 2007. Phenol content was measured by using Folin–Ciocalteau method (Mahdavi, Nikniaz, Rafraf, & Jouyban, 2010). Antioxidant activity was determined by DPPH radical scavenging method as determined by Sochor et al., 2010 and Stajcic et al., 2012.

%DPPH radical scavenging activities = [(A_0_ − A_1_/A_1_) × 100].

where *A*_0_ is the absorbance of the control (DPPH).

*A*_1_ is the absorbance of the sample.

### 2.5 Sensory analysis

Ten untrained panelists (college students and staff) were involved to assess the sensory properties according to 9 point hedonic ranking scale. The selection criterion was the students researching on yacon and staff familiar with the original taste of yacon (regular consumers). The age composition of the panelist was 70% (19–25 years) and 30% (35–40 years), whereas the gender composition was 40% male and 60% female. The sensory scores included: Like extremely = 9, Like very much = 8, Like moderately = 7, Like slightly = 6, Neither like nor dislike = 5, Dislike slightly = 4, Dislike moderately = 3, Dislike very much = 2, and Dislike extremely = 1. The panelists were also asked to make some recommendations/comments about the samples on the score sheets used for the sensory evaluation. Each sample was given a random 3 digit code in their labels, and water was provided to clear the throat between sample evaluations. The sensory analysis of freshly prepared juice, as well as the stored juices on refrigerated condition (fermented and nonfermented juice stored for 25 days), was carried out for color, flavor, mouthfeel, and overall acceptance (Garcia‐Gomez, Romero‐Rodriguez, Vazquez‐Oderiz, Munoz‐Ferreiro, & Vazquez, 2019).

### 2.6 Statistical analysis

The analysis was carried out in triplicate. The data were statistically analyzed using SPSS Edition 20 and Microsoft excel 2007. Data were analyzed for significant difference by ANOVA at 5% level of significance using Tukey test.

### 2.7 Experimental design

The experiment is carried out in a completely randomized design with two treatments and three replicates for chemical and microbial analysis.

## 3 RESULT AND DISCUSSION

### 3.1 Changes during fermentation and storage

The results showed decrease in pH while acidity increased during fermentation at 37°C for 24 hr (Table 1). The result is in accordance with Pedreschi et al., 2003 where the pH of yacon extracts decreased from 6.5 to 4.85 on *L. acidophilus* fermentation in anaerobic condition 37°C for 24 hr. Similarly, there was significant growth in biomass. The growth in biomass may be because of the presence of yacon FOS which is utilized by the *L. acidophilus* under anaerobic conditions (Pedreschi et al., 2003).

**Table 1:** Changes during fermentation

Parameters	Initial	Fermentation (24 hr)
^ο^Brix	8 ± 0.05	7.8 ± 0.05
pH	6.80 ± 0.02	4.58 ± 0.03
Acidity (%)	0.07 ± 0.00	0.61 ± 0.01
Lactobacillus count (log cfu/ml)	7	8.59

Note

Values are mean ± standard deviation obtained from triplicate data.

### 3.2 Physicochemical changes upon storage

**Figure 1:**
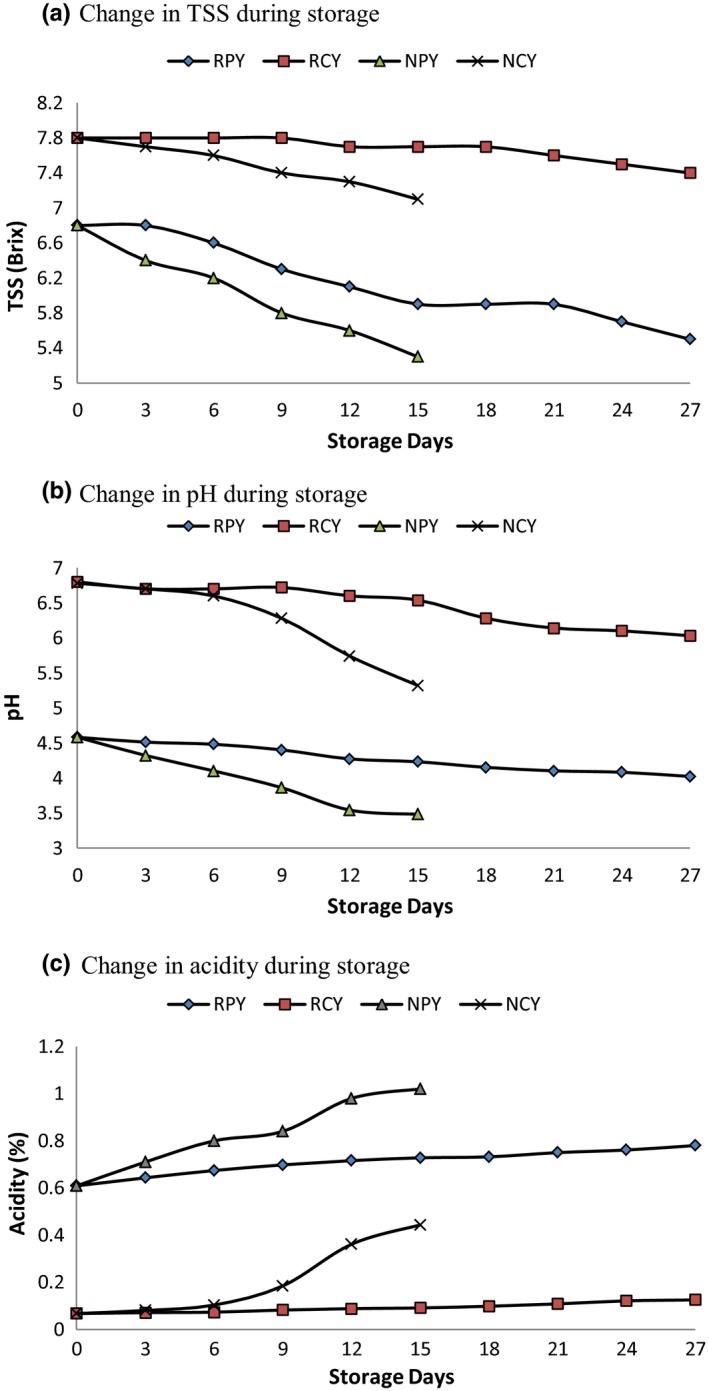
Change in (a), TSS (b), pH and (c), acidity during storage for Probiotic and nonfermented juice upon storage at refrigerated and nonrefrigerated conditions

The change in total soluble solid (TSS), pH, and acidity during storage was monitored in the space of 3 days and shown in Figure 1a‐c, respectively.

The decrease in pH and increase in acidity were significant in probiotic juice as compared to nonfermented juice in both refrigerated and nonrefrigerated conditions. For fermented juice RPY, and NPY, the pH decreased from 4.58 to 4.02 ± 0.02 and 3.48 ± 0.02 after 27 and 15 days of storage, respectively. However, for nonfermented sample pH remained quite stable and decreased from 6.78 ± 0.02 to 6.03 ± 0.02 and 5.32 ± 0.02, respectively, after 27 and 15 days of storage. Similarly, the initial acidity of samples RPY and NPY (Probiotic sample) was 0.609 ± 0.02 which gradually increased to 0.80 ± 0.02 and 1.02 ± 0.02 after 27 and 15 days of storage, respectively. However, for nonfermented sample RCY and NCY, the acidity was initially 0.067 ± 0.02 which increased to 0.125 ± 0.02 and 0.443 ± 0.02, respectively, after 27 and 15 days of storage.

Similar results were reported for acidity and pH of fermented cashew apple juice as revealed by Pereira, Almeida, Jesus, Costa, & Rodrigues, 2012, and lactic acid content increased during storage, indicating that *L. acidophilus* can produce acid both at room temperatures and refrigerated condition while the higher increase in acidity was at room temperature than refrigerated temperature. (Abbo, Olurin, & Odeyemi, 2006).

### 3.3 Microbial changes upon storage

**Figure 2:**
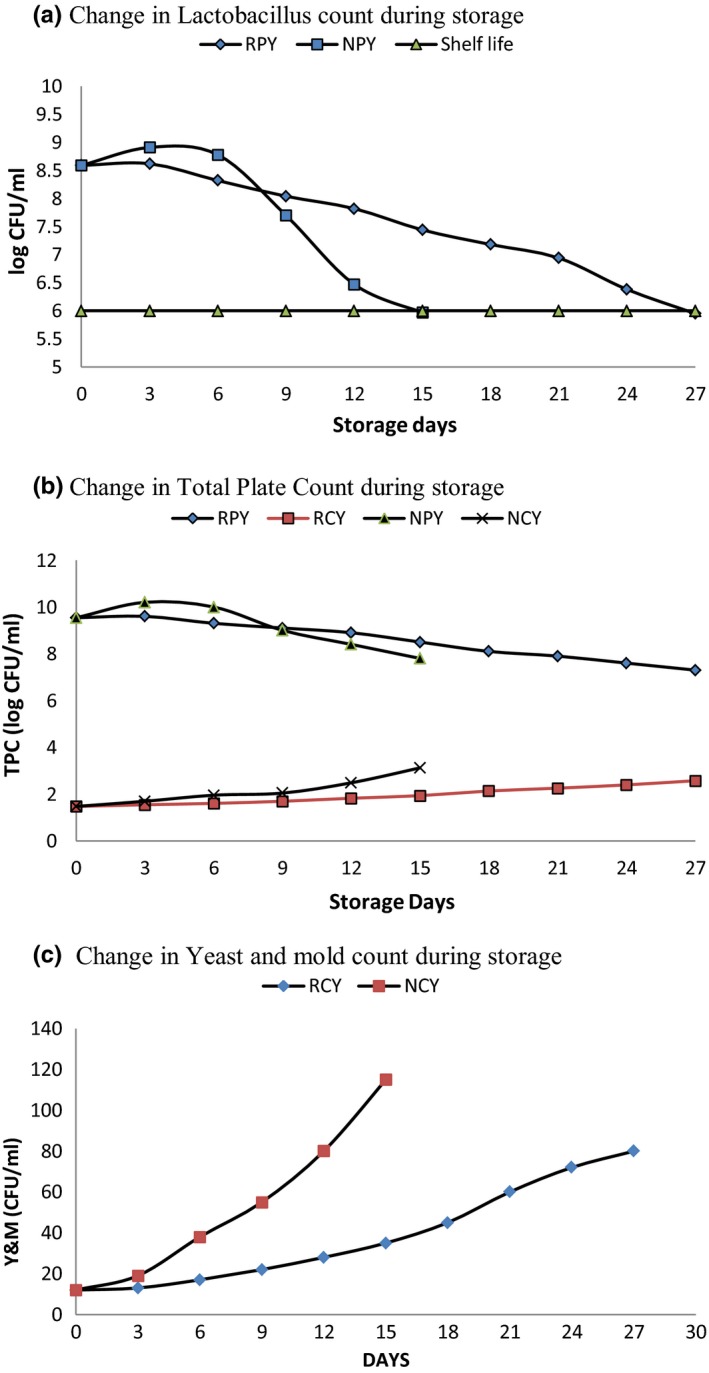
Change in (a), Lactobacillus count (b), total plate count and (c), yeast and mold count during storage for probiotic and nonfermented juice upon storage at refrigerated and nonrefrigerated conditions

The change in Lactobacillus count, TPC and yeast, and mold during storage was monitored in the space of 3 days and shown in Figure 2a‐c, respectively.

The initial LAB count of sample RPY and NPY was decreased from 8.59 to 5.97 log CFU/ml and 5.95 log CFU/ml after 27 and 15 days of storage, respectively. The viability of the probiotic microorganism was higher than whey and pineapple beverage fermented with *L. acidophilus* (Shukla, 2012). This may be due to the utilization of yacon FOS by probiotic microorganism under anaerobic conditions resulting in excessive growth and prolonged viability in the product. So, as the viability of microorganisms remained around 6 log CFU/ml for 15 days at normal room condition and 27 days at refrigerated condition, the shelf life of the juice for its probiotic property can be implied as 15 and 27 days at normal room and refrigerated condition, respectively.

Similarly, total plate count (TPC) of the nonfermented sample was 1.47 log CFU/ml which increased to 2.57 and 3.17 log CFU/ml after storing for 27 and 15 days. However, TPC of the fermented sample was higher initially 9.54 log CFU/ml and decreased to 7.3 and 7.8 log CFU/ml during the storage time.

There was no growth of yeast and mold in probiotic juice and for nonprobiotic, and the growth was in the minimal count and hence expressed in CFU/ml. Lack of oxygen during storage along with blanching and pasteurization during juice extraction might be the main reason for low yeast and mold count and growth during storage. The coliform count was zero for all products indicating hygienic conditions applied during product development. As there was no significant growth of yeast and mold and coliform during the storage time, the fermented and nonfermented juice can be consumed as normal juice even after storage for 15 days at normal room conditions and 27 days at the refrigerated condition.

### 3.4 Physicochemical analysis of yacon juice

The mean score for the physicochemical properties of all the samples and statistical analysis (ANOVA) for the values obtained were presented in Table 2.

**Table 2:** Chemical composition of yacon juice

Parameters		INI	RPY	NPY	RCY	NCY
pH		6.8 ± 0.04^a^	4.02 ± 0.01^b^	3.48 ± 0.1^c^	6.03 ± 0.045^d^	5.38 ± 0.04^e^
Total	soluble	8 ± 0.26^a^	5.5 ± 0.26^b^	5.3 ± 0.10^b^	7.4 ± 0.26^c^	7.1 ± 0.10^c^
Solid	(°Brix)					
Titrable acidity (%)		0.067 ± 0.05^a^	0.80 ± 0.06^b^	1.02 ± 0.04^c^	0.12 ± 0.05^a^	0.44 ± 0.02^e^
Reducing Sugar(g/100 g)		4.23 ± 0.04^a^	1.45 ± 0.03^b^	1.24 ± 0.05^c^	4.29 ± 0.02^ad^	4.35 ± 0.02^d^
Total (g/100 g)	Sugar	6.34 ± 1.22^a^	2.83 ± 0.15^b^	2.54 ± 0.07^c^	5.9 ± 0.06^a^	5.48 ± 0.15^e^
Clarity (%)		26.33 ± 0.98^a^	15.49 ± 0.30^b^	ND	19.41 ± 0.24^c^	ND

Note

Values are the mean ± standard deviation obtained from triplicate data. Values in the row having different superscript are significantly different.

Abbreviations: INI, Initial freshly prepared juice; NCY, Normal Control Yacon; NPY, Normal Probiotic Yacon; RCY, Refrigerated Control Yacon; RPY, Refrigerated Probiotic Yacon.

* ND = Not Determined.

The significant reduction of sugar content in probiotic added juice is due to the fermentation process. During fermentation homofermentative, Lactobacillus is responsible for the conversion of fermentable sugar into lactic acid resulting in the reduction of the reducing sugar. Surprisingly, for normal juice, the reducing sugar value showed a slight increase over the storage period. Wong, 2003 and Bibas Bonet et al.., 2010 studied the effect of storage on FOS of yacon and the result showed the conversion of FOS to glucose, fructose, and sucrose at a temperature higher and lower than 10°C which might be the reason for the increase in reducing sugar value.

### 3.5 Bioactive compounds and antioxidant property

**Figure 3:**
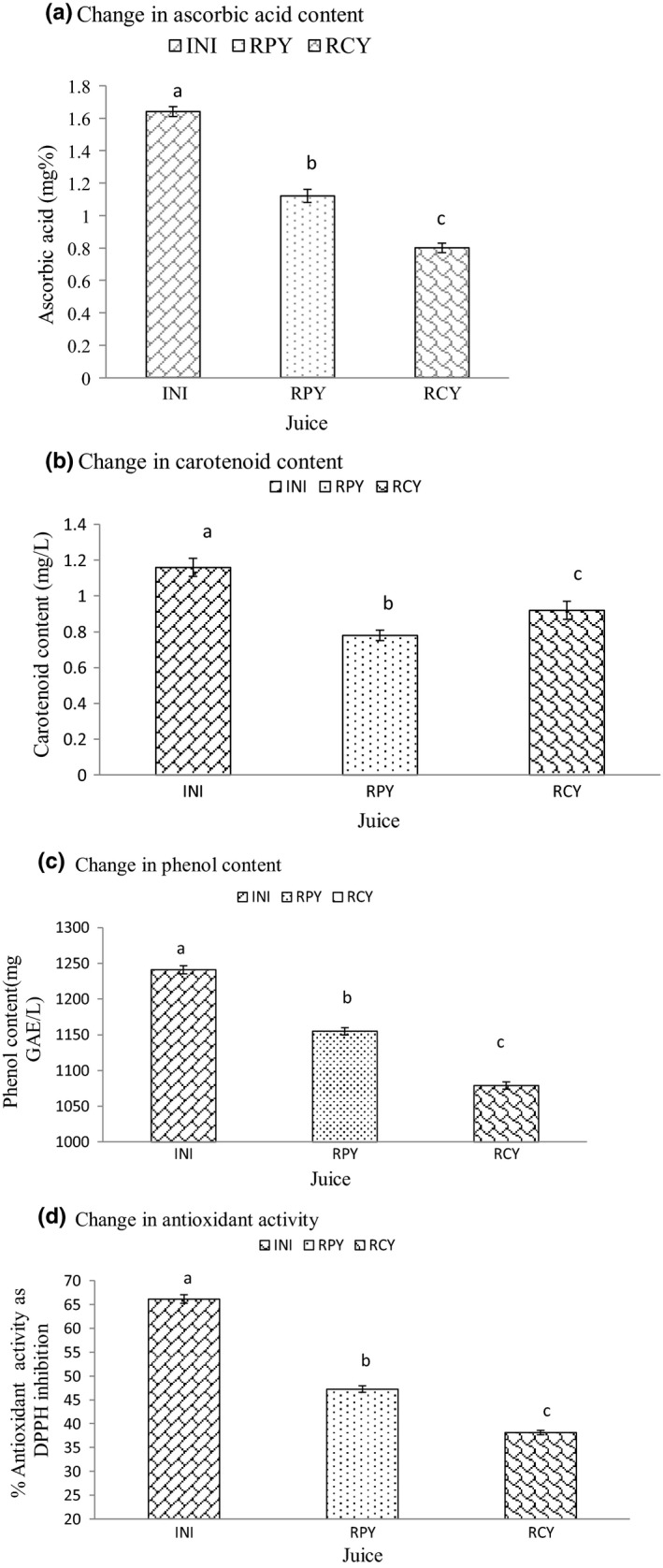
Change in (a), ascorbic acid (b), carotenoid content and (c), total phenol content and (d), antioxidant activity during storage for fermented and nonfermented juice upon storage at refrigerated condition

The change in the amount of ascorbic acid, carotenoid content, phenol content, and antioxidant activity from the freshly prepared juice to fermented and control juice stored in refrigerated condition for 27 days is shown in Figure 3a‐d, respectively.

The bioactive components were higher in the freshly extracted initial juice. Upon storage, the bioactive components decreased. However, the decrease was less severe for fermented juice as compared to nonfermented juice except for carotenoid value. Similar results were also seen during the storage of fermented (*Lactobacillus plantarum*) and nonfermented pomegranate juice stored for 30 days (Filannino et al., 2013). Thus, the above result indicates fermentation of juice by *Lactobacillus acidophilus* preserves the bioactive component of the yacon juice.

**Figure 4:**
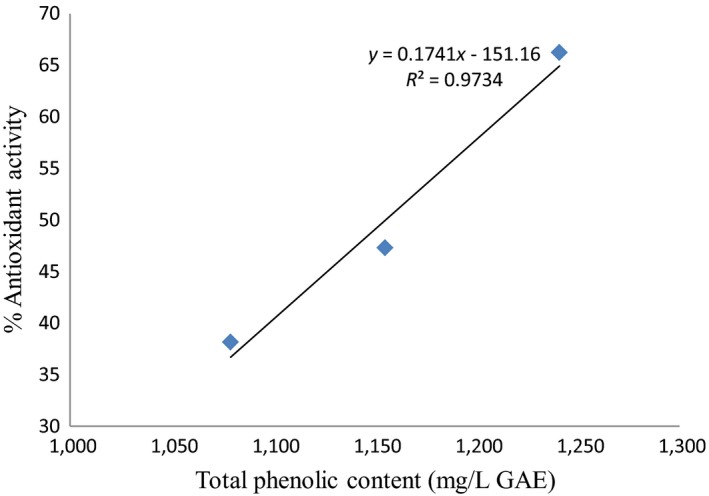
Correlation between antioxidant activity and total phenolic content of yacon juice

The antioxidant activity showed a similar trend. The total polyphenolic content of yacon juice had a positive correlation with the antioxidant activity as shown in Figure 4. The reduction in both the cases was higher in nonfermented juice as compared to the fermented one. The result presented is in agreement with Pereira et al., 2012 who evaluated the effect of lactic acid fermentation on cashew apple juice. The less intense decrease in fermented juice may be due to increase in the concentration of antioxidant compounds such as polyphenol, flavanoid, and beta carotene during fermentation by lactic acid bacteria (Hernandez et al., 2007; Wu, Su, & Cheng, 2011).

### 3.6 Sensory analysis

**Figure 5:**
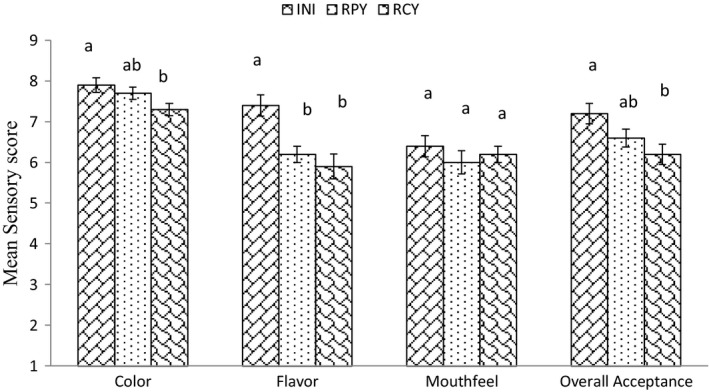
Consumer preference for different probiotic juice

The sensory analysis result is shown in Figure 5.

A comparative sensory analysis between INI (fresh juice), RCY, and RPY (stored for 25 days) showed no significant difference at 5% level of significance. The acceptance for all the juice was very good even after 25 days of storage.

## 4 CONCLUSION

The result concludes that yacon can act as a substrate for the growth of probiotic microorganisms and the storage of yacon as synbiotic juice is beneficial in both sensory and nutritional aspects. The low yeast and mold count indicated synbiotic juice can be continued to be consumed as a normal juice even after the viability of the lactic organism decreased.

The fermentation provided a good preservative effect on phenolic content and antioxidant activity of yacon juice which confers the nutritional benefits of the functional food prepared.

## CONFLICT OF INTEREST

The authors declare that they do not have any conflict of interest.

## ETHICAL APPROVAL

This study does not involve any human or animal testing.

## INFORMED CONSENT

Written informed consent was obtained from all participants.
